# Monetary Value of Diet Is Associated with Dietary Quality and Nutrient Adequacy among Urban Adults, Differentially by Sex, Race and Poverty Status

**DOI:** 10.1371/journal.pone.0140905

**Published:** 2015-11-04

**Authors:** May A. Beydoun, Marie T. Fanelli-Kuczmarski, Allyssa Allen, Hind A. Beydoun, Barry M. Popkin, Michele K. Evans, Alan B. Zonderman

**Affiliations:** 1 Laboratory of Epidemiology and Population Sciences, National Institute on Aging, Intramural Research Program, NIA/NIH/IRP, Baltimore, Maryland, United States of America; 2 Department of Behavioral Health and Nutrition, University of Delaware, Newark, Delaware, United States of America; 3 University of Maryland, Baltimore County, Baltimore, Maryland, United States of America; 4 Graduate Program in Public Health, Eastern Virginia Medical School, Norfolk, Virginia, United States of America; 5 Department of Nutrition and Carolina Population Center, University of North Carolina at Chapel Hill, Chapel Hill, North Carolina, United States of America; Swinburne University of Technology, AUSTRALIA

## Abstract

**Objective:**

The association between monetary value of the diet (MVD, $/day) with dietary quality was examined using a large sample of urban US adults, differentially by socio-demographic factors.

**Methods:**

This was a cross-sectional study of 2,111 participants, aged 30–64y, using data from the Healthy Aging in Neighborhoods of Diversity across the Life Span Study. Dietary quality indices included Healthy Eating Index–2010 (HEI–2010) and Mean Adequacy Ratio (MAR), (two 24-hr recalls). A national food price database was used to estimate MVD. Multiple linear/logistic regression analyses were conducted stratifying separately by sex, race and poverty status.

**Results:**

Women had significantly higher HEI-2010 scores than men (43.35 vs 41.57 out of 100, respectively), whereas MAR scores were higher for men (76.8 vs 69.9, out of 100), reflecting energy intake gender differentials. Importantly, a $3/day higher MVD (IQR: $3.70/d (Q1) to $6.62/d (Q4)) was associated with a 4.98±0.35 higher total HEI-2010 and a 3.88±0.37 higher MAR score, after energy-adjustment and control for key confounders. For HEI-2010 and MAR, stronger associations were observed among participants above poverty and among women, whilethe MVD vs. HEI-2010 association was additionally stronger among Whites. Sex and poverty status differentials were observed for many MAR and some HEI-2010 components.

**Conclusions:**

Despite positive associations between measures of dietary quality and MVD, particularly above poverty and among women, approaching compliance with the Dietary Guidelines (80 or more for HEI-2010) requires a substantially higher MVD. Thus, nutrition education may further improve people’s decision-making regarding food venues and dietary choices.

## Introduction

Non-communicable diseases such as cardiovascular disease and cancer are associated with a short list of modifiable risk factors. These risk factors include obesity resulting from unhealthful diet with excessive energy and physical inactivity, tobacco use, excessive alcohol consumption, uncontrolled high blood pressure, and hyperlipidemia.[1] A present challenge when studying diet-disease relationships is the ability to distinguish between healthful vs. unhealthful dietary patterns due to measurement error[2] and the salient multiplicity in methodological approaches used [3, 4]. Overall diet quality can be evaluated through comparison with a variety of indices or scoring methods based on food groups or nutrients or a combination of the two. Two commonly used non-data driven diet quality indices in the United States are: (1) The Healthy Eating Index-2010 (HEI-2010), a primarily energy-adjusted food-group based index which assesses conformity to the most recent Dietary Guidelines for Americans [3–7], and (2) The Mean Adequacy Ratio (MAR), a purely micronutrient-based diet quality measure, comparing the mean intake of a nutrient intake to its Recommended Dietary Allowance (RDA).(8)

Consumption of a healthful diet consistent with the Dietary Guidelines for Americans will most likely require many individuals to alter food choice. Food choices depend on individuals’ health status, personal preferences, cultural influences but perhaps more importantly socio-economic constraints, such as income. Food expenditure is often the part of total household expenditure that is subjected to substantial cuts in times of economic strains.[8] It has been hypothesized that under financial constraints, people often gravitate around less expensive foods. [8] Paradoxically, reducing diet costs may lead to the selection or increased use of energy-dense foods, such as refined grains, foods high in added sugars and/or fats, which can lower overall dietary quality.[9–13]

Uncovering the relationships between food prices in the environment or household expenditure on food and food consumption patterns have been a focus of current research. [14–19] Additionally, the direct association between dietary patterns and the monetary value of the diet (MVD) among individuals has also been examined.[20–50] While the majority of research documents a direct relationship of the MVD with diet quality and with the consumption of lower energy density foods, [20–28, 30–35, 37–39, 41–44, 47, 48, 51] some study results did not corroborate these findings.[29, 36, 40, 45, 46].

To date, no study has explored differential associations between MVD and dietary quality, measured with the HEI-2010 and MAR, while examining differential effects by sex, race and poverty status and focusing on a low-income US urban adults population. The objective of this study was to examine not only differentials across sex and race/ethnicity groups as was done in the context of the US population,[51] but also across income groups using a large sample of low-income urban US adults.

## Materials and Methods

### Database and participants

Initiated in 2004, Healthy Aging in Neighborhoods of Diversity across the Life Span (HANDLS) study is an ongoing prospective cohort study focused on the cardiovascular and cognitive health of a socioeconomically diverse sample of African Americans and Whites (30–64 years old at baseline), living in Baltimore, Maryland. The sample selection methodology is described elsewhere. [52] In brief, using area probability sampling of thirteen neighborhoods, phase 1 consisted of screening, recruitment, first dietary recall, and household interviews, whereas phase 2 consisted of the second dietary recall and examinations in mobile Medical Research Vehicles (MRV). Cross-sectional data from participants examined in the baseline wave 1, 2004–2009, were used in this study.

Written informed consent was obtained from all participants following their access to a protocol booklet in layman's terms and a video describing all procedures and future re-contacts. Ethical approval of the HANDLS study was obtained from the MedStar Institutional Review Board.

The present sample consisted of 2,111 individuals who completed two days of 24-hour dietary recalls. Participants completing only phase 1 recall (n = 1,235) were excluded since 2 dietary recalls provide a better indicator of typical food intake. An additional 374 individuals had missing data on both dietary recall data and phase 2-measured covariates including literacy. There were no statistical differences in the distributions of demographic data or energy and nutrient profiles or in the distributions of total HEI-2010 scores between participants who completed one (N = 1,235) or both days of dietary recall (N = 2,111). Thus, the study sample was considered representative of the entire HANDLS baseline sample, with 510 White women, 683 African-American women, 383 White men and 535 African-American men; and with overall 906 living below poverty and 1,205 above the federal poverty line (≥125% of the poverty income ratio, PIR).

The HANDLS study participants represents a unique understudied population in US nutrition surveys. It is important to recognize that although the HANDLS sample is restricted to adults residing with the confines of Baltimore city, the results from HANDLS can be generalized to urban adults in the same age range residing in cities with similar population density and racial distributions as Baltimore. These cities include Atlanta, GA; Bridgeport, CT; Bridgeton, NJ; Buffalo, NY; Camden, NJ; Carson, CA; Chicago, IL; Cleveland, OH; Detroit, MI; Harrisburg, PA; Hartford, CT; Oakland, CA; Springfield, MS and Trenton, NJ.” [Lepkowski J. HANDLS Generalizibility, 2010 and HANDLS Principle Cities Clusters Analysis, 2011, unpublished internal National of Institutes on Aging documents]

### Dietary Collection Method

The US Department of Agriculture (USDA) Automated Multiple Pass Method, a computerized structured interview, was used to collect both 24-hour dietary recalls.[53] Measurement aids included measuring cups, spoons, ruler, and an illustrated Food Model Booklet that assisted participants in estimating accurate quantities of foods and beverages consumed. Both recalls were administered in-person by trained interviewers, 4 to 10 days apart, during the study period (2004–2009). Dietary recalls were coded by trained nutrition professionals using the statistical software Survey Net,[54] matching foods consumed with 8-digit codes in the Food and Nutrient Database for Dietary Studies version 3.0.[55]

### Key outcome measures

#### Healthy Eating Index – 2010

The National Cancer Institute website on Applied Research provided a series of computational steps for the HEI-2010 component and total scores as well as a statistical code for 24-hour recalls.[56] Detailed description of the procedure used is available on the HANDLS website.[57] Total and component HEI-2010 scores were calculated for each recall day (day 1 and day 2) and then averaged to obtain the mean HEI-2010 total and component scores for both days combined.

#### Mean Adequacy Ratio (MAR)

Nutrient-based diet quality was determined based on models published by Raffensperger and colleagues[58] and Murphy and colleagues,[59]. Dietary intakes of calcium, magnesium, phosphorus, Vitamin A, Vitamin D, Vitamin C, Vitamin E, Vitamins B_6_ and B_12_, folate, iron, thiamin, riboflavin, niacin, copper, and zinc were used as the basis for diet quality. The RDAs of these 16 vitamins and minerals were used to determine the nutrient adequacy ratio (NAR), using the following formula: NAR = Subject’s daily intake of nutrient divided by the RDA of nutrient. An adjustment of an additional 35 mg vitamin C was applied to the RDA for participants who were current smokers.[60] The NAR of each nutrient was then converted to a percent, and percentages were truncated to 100%. [59] The total quality of the diet was then calculated from the NARs to form a mean adequacy ratio (MAR) using the following formula: MAR = Sum of all 16 nutrient NARs divided by 16.

### Key explanatory measure: Monetary value of diet estimation (MVD)

Diet cost per 100 grams was estimated using the CNPP Food Prices Database, 2003–04, which provides the average national prices of nearly 4,600 foods in an “as-consumed” form for that year.[61] Foods included are those consumed alone (apples, carrots, etc.), prepared using a recipe with different ingredients (noodle casserole, chicken pot pie, etc.), as well as commercially prepared foods (frozen dinners, canned soups, etc.). A distinction was made between food “as-consumed” and food “as-purchased,” with the former accounting for weight changes related to cooking while excluding refuse, namely peels of vegetables and meat bones. The CNPP database was ultimately generated by combining information from four data sets: The National Health and Nutrition Examination Survey (NHANES), 2003–04; the USDA Food and Nutrient Database for Dietary Studies (FNDDS) version 2.0; the National Nutrient Database for Standard Reference (Release 20); and the Nielsen Homescan ™ Consumer Panel. More details are provided about the methodology elsewhere.[62] This database provided food prices per 100 grams at the national level for about 4,600 food codes elicited in the NHANES 2003–04 and facilitated MVD per day estimates among HANDLS baseline participants with two days of dietary recall.

Steps **A-C** were needed to compute MVD in HANDLS: **(A)** The multiple-record HANDLS data were merged with NHANES 2003–04 food price database.[61] This step allowed us to determine which food codes in HANDLS required imputation with food prices not reported in the NHANES 2003–04 database. **(B)** By collapsing data in HANDLS per food code, the count of food codes requiring imputation were determined. In our data, 312 food codes needed to be imputed, with a small proportion of alcoholic beverages (11%) and the remaining missing codes spanning all other food groups, with the possible exception of eggs. (3) **(C)** Manual imputations were made by replacing missing data on prices/100 grams with a nearest neighbor food code with non-missing price/100g that appeared to be similar enough to food code with missing data. **(D)** Alcoholic beverages, for which prices were all missing in the NHANES 2003–04 database, were estimated using the Quarterly Food Away from Home database.[63] Consequently, prices per 100 g for the year 2003–04 were estimated as: Beers and ales: $0.089791/100g; Cordials and liqueurs: $0.59857/100g; Cocktails: $0.59857/100g; Wines: $0.259936 /100g; Distilled liquors: $0.59857/100g. In this computation, 100 gram of beverage was equivalent to 3.3814 fl oz; **(E)** It is worth noting that the food price database used does not include the added costs associated with the preparation and service of foods and beverages consumed in outside the home in 2004 which contributed to about 39% of food consumed by US adults, ranging from 30% for the poorest to 44% for the wealthiest population groups. (65) Thus, although prices in the database used were estimated as if the food was prepared and consumed at home, a measure of % energy consumed at home as purchased from grocery stores averaged over the two days of recall was obtained to control for price inflation due to consuming foods away-from-home. A previous study had adjusted away-from-home prices by estimating that they were 1.4–2.0 the price of the same food prepared at home.[64] However, we determined that the approach of adjusting models for % energy consumed away-from-home was less susceptible to assumptions about the exact inflation factor to use. No other adjustment was made in terms of inflation since the food price database used was limited to the years 2003–04.

### Covariates

Several covariates were considered as potential confounders including age, sex, race (White *vs*. African American), marital status (married *vs*. unmarried), completed years of education (<High School (HS); HS and >HS), literacy (WRAT-3 total score), poverty status, a design-based binary variable in HANDLS based on poverty income ratio (PIR<125%: below poverty; PIR≥125%: above poverty), and current smoking status (0: “never or former smoker” and 1 “current smoker”). The reading subtest of the Wide Range Achievement Test-3^rd^ Edition (WRAT-3), a widely validated measure of literacy, assessed participants’ ability to recognize and name letters and words, with a total score computed as “total correctly pronounced letters + total correctly pronounced words”.[65] As stated earlier, models were also adjusted for % energy consumed at home (food items purchased at grocery stores) to adjust for price inflation in away-from-home settings. Energy intake (kcal/d) from the total diet was adjusted for in all regression models.

### Statistical analysis

All analyses were conducted using Stata release 13.0 (StataCorp, College Station, TX).[66] First, socio-demographic characteristics were assessed by sex and MVD tertiles. Mean differences between groups were tested using a non-parametric test for trend, while relationships among categorical variables were evaluated with χ^2^ tests.

Second, multiple ordinary least square (OLS) regression models with 2010-HEI total score as the target outcome were conducted to test the association between the MVD exposure (per 1$/day) and dietary quality, controlling for potential confounders listed under covariates section. The same approach was carried out with MAR and its individual components. Comparing the uppermost tertile of each component of the 2010-HEI to the lower two tertiles combined as a referent category, a set of multiple logistic regression models were conducted to test the association between MVD as an exposure and the likelihood of “better dietary quality” on each of those components.

To account for potential selection bias in regression models (due to the non-random selection of participants with complete data from the target study population), a 2-stage Heckman selection model was constructed,[67] using a probit model to obtain an inverse mills ratio at the first stage (derived from the predicted probability of being selected, conditional on the covariates in the probit model, mainly baseline age, sex, race, poverty status and education), as was done in an earlier study.[68]

In all regression analyses listed above, the moderating effect of poverty status was tested by adding interaction terms to the multivariable models and stratifying the models by poverty status. Similarly, moderation by sex and race was also tested with two-way interaction terms entered into two separate models and a separate stratification by each of those two socio-demographic variables. Type I error was set at 0.05 for main effects and 0.10 for interaction terms due to the latter’s reduced statistical power compared to the former.[69]

## Results

### Study sample characteristics by monetary value of diet (MVD) tertile and sex

The distribution of selected baseline characteristics by MVD tertile is presented in **Table 1**. Overall, energy intake was linearly and positively related to MVD (P-trend<0.001).

**Table 1:** Baseline characteristics of selected HANDLS sample by tertile of monetary value of diet (MVD) (N = 2111).

	MVD tertiles ($/d)			
	T1	T2	T3	P-trend ^a^
	0.9–4.1	>4.1–6.0	>6.0–31.0	
**Monetary value of diet and energy intakes**				
Monetary value of diet, $/day (X ± SE)	3.11±0.03	5.03±0.02	8.40±0.10	<0.001
Energy intake, kcal/d (X ± SE)	1388±18	1939±22	2711±44	<0.001
**Socio-demographic and SES variables**				
Sex, % male	33.4	41.5	55.6	<0.001
Age, yrs. (X ± SEM)	49.0±0.4	48.8±0.4	47.4±0.3	0.27
African-American, %	60.2	57.4	55.5	0.19
Income, % (<125% PIR)	46.3	43.5	39.0	0.020
Education, yrs. completed (X ± SE)	11.8±0.1	12.6±0.1	13.1±0.1	<0.001
<HS	10.5	5.0	4.7	
HS	63.1	60.1	53.3	
>HS	26.4	34.9	42.0	
Literacy, WRAT-3 score (X ± SE)	39.4±0.4	42.2±0.4	42.6±0.4	<0.001
<36, %	29.8	20.0	21.3	<0.001
37–40, %	18.0	14.6	13.1	
41–46,%	29.3	26.3	23.2	
≥47,%	22.9	39.2	42.4	
% Unemployed in last month, yes	42.5	35.4	32.6	<0.001
% Unemployment in last month, missing	19.3	17.3	15.4	
**Drug and tobacco use**				
Any drug, current user, %	12.9	15.6	19.4	0.002
Any drug, missing, %	4.7	6.3	7.3	
Tobacco, current user, %	48.3	40.3	45.2	0.014
Tobacco, missing, %	4.7	6.3	7.3	
**Self-rated health**				
Poor, %	4.7	4.7	4.0	0.048
Average, %	24.7	19.9	17.8	
Good, %	37.6	43.1	40.5	
Very good, %	25.1	25.0	28.3	
Excellent %	7.8	7.3	9.4	
**% energy from grocery stores**				
100%	36.3	27.6	23.0	<0.001
50–99.9%	51.4	59.0	63.0	
≤50%	12.4	13.5	12.0	

^a^ P-value for trend (continuous variables) or P-value from χ2 test (categorical variables). PIR = Poverty Income Ratio; SE = Standard Error; WRAT-3 = Wide Range Achievement Test, version 3.

While MVD was not associated with race or age, percent male, percent above poverty, educational attainment and literacy were all positively associated with MVD, while unemployment rate was inversely related to MVD. (P-trend<0.001), Overall, the likelihood of energy intake to be completely derived from grocery store foods was inversely related to MVD tertiles, indicating that grocery store foods are less expensive than other sources. Moreover, mean MVD was significantly higher among men compared to women (Mean±SE: 6.2±0.1 vs. 5.0±0.1, p<0.001), with no detectable differences by race or poverty status (S1 Table). Other interesting patterns of differences by sex, race and poverty status are shown in S1 Table, including higher education, income and literacy coupled to lower unemployment among Whites compared to African-Americans; higher drug and tobacco use among men, and a lower likelihood of self-rated health as “Excellent” or “Very good” among African-Americans compared to Whites.

**Fig 1:**
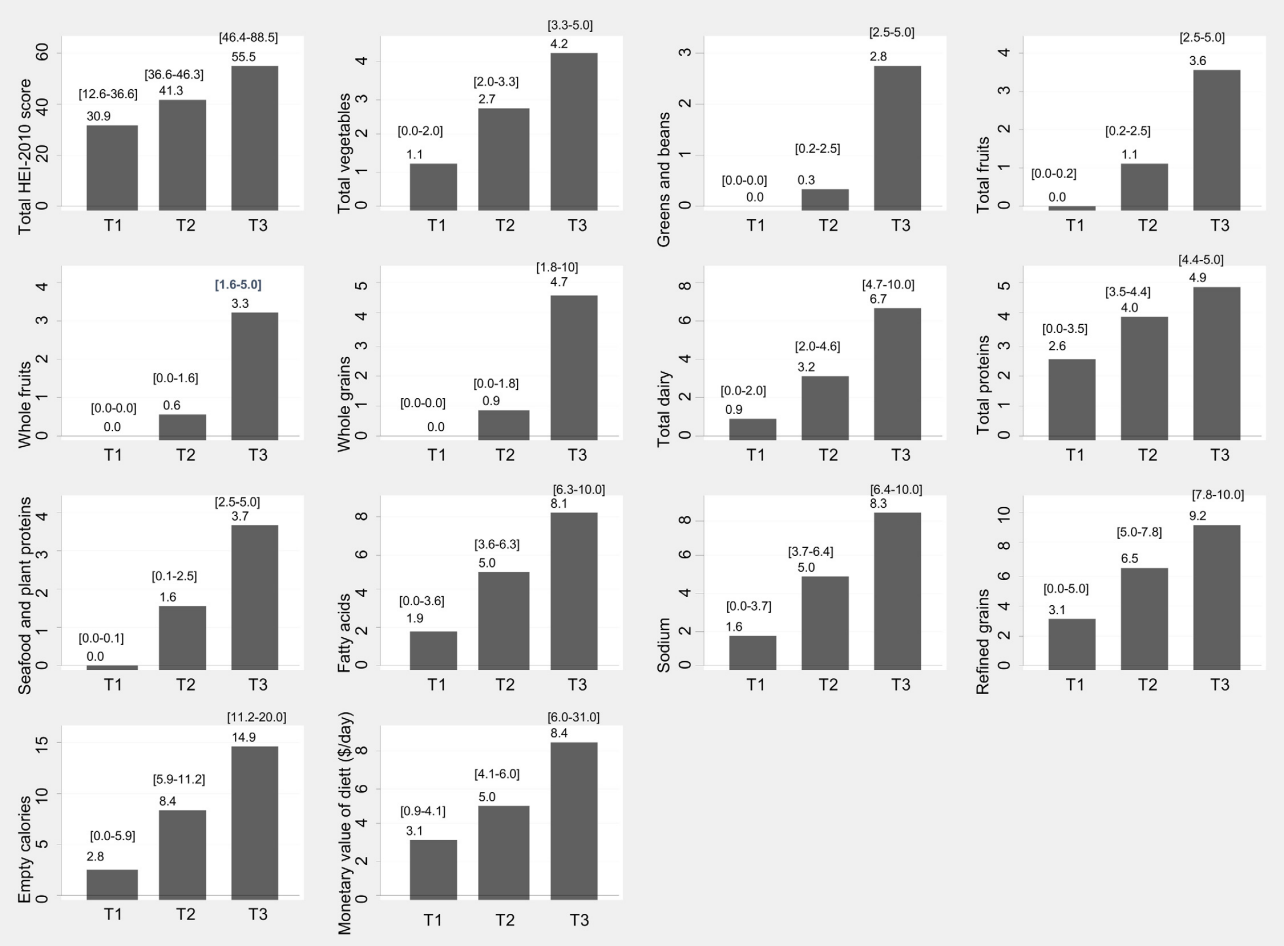
Means and [ranges] of HEI-2010 (total score and components) and of monetary value of diet (MVD, $/day) by their respective tertiles; HANDLS study, wave 1.

**S1 Fig** presents overall means of HEI components and **S2 Fig** shows means of NAR and MAR. **Fig 1** also shows the mean values and ranges for respective tertiles of HEI components.

### Dietary quality by diet cost tertile and sex

**Table 2** shows the bivariate association between HEI-2010 (total score and components) and tertiles of MVD. Among both men and women, mean HEI-2010 total score increased in a linear fashion with each MVD tertile (P-trend<0.001), with an approximate 6-point gain among men between T_1_ and T_3_ and a 9-point gain among women. This change was also observed for most HEI-2010 components with a clear increasing linear trend among both men and women. However, the increase in HEI-2010 did not indicate that the diet was even close to the optimal score of 100

**Table 2:** HEI-2010 total score and components^a^ for HANDLS participants by monetary value of diet tertile and sex.

			**Men (n = 918)**			
			**MVD tertiles ($/d)**			
		**All**	**T1**	**T2**	**T3**	
			**0.9–4.1**	**>4.1–6.0**	**>6.0–31.0**	
	**Max score**	**X±SEM**	**X±SEM**	**X±SEM**	**X±SEM**	**P-trend ^a^**
HEI-2010 total score	100	41.57±0.35^b^	37.86±0.61	41.36±0.60	43.96±0.56	<0.001
Total vegetables	5	2.53±0.04^b^	2.26±0.09	2.53±0.08	2.68±0.06	0.004
Greens and beans	5	0.85±0.04^b^	0.72±0.08	0.74±0.07	1.02±0.07	<0.001
Total fruit	5	1.47±0.05^b^	0.97±0.10	1.52±0.10	1.72±0.08	<0.001
Whole fruit	5	1.09±0.05^b^	0.61±0.08	1.09±0.09	1.38±0.09	<0.001
Whole grains	10	1.63±0.07^b^	1.48±0.14	1.60±0.12	1.76±0.12	0.010
Dairy	10	3.62±0.08	3.41±0.17	3.73±0.15	3.67±0.13	0.250
Total protein foods	5	4.32±0.03^b^	4.20±0.07	4.29±0.05	4.43±0.04	0.046
Seafood and plant proteins	5	1.53±0.05^b^	1.11±0.10	1.37±0.09	1.89±0.08	<0.001
Fatty acids	10	4.94±0.09	4.74±0.18	5.00±0.15	5.02±0.13	0.250
Sodium	10	4.96±0.10	5.08±0.19	5.19±0.16	4.71±0.15	0.070
Refined grains	10	6.19±0.09	5.64±0.19	6.24±0.16	6.48±0.14	<0.001
Empty calories	20	8.44±0.18	7.66±0.34	8.06±0.30	9.20±0.28	<0.001
			**Women (n = 1193)**			
			**MVD tertiles ($/d)**			
		All	**T1**	**T2**	**T3**	
			**0.9–4.1**	**>4.1–6.0**	**>6.0–31.0**	
		X±SEM	X±SEM	X±SEM	X±SEM	P-trend ^a^
HEI-2010 total score	100	43.35±0.35	39.12±0.48	44.91±0.58	47.66±0.70	
Total vegetables	5	2.78±0.04	2.49±0.06	2.84±0.07	3.14±0.07	<0.001
Greens and beans	5	1.04±0.04	0.75±0.06	1.03±0.07	1.50±0.09	<0.001
Total fruit	5	1.64±0.05	1.31±0.07	1.75±0.08	2.00±0.10	<0.001
Whole fruit	5	1.28±0.05	0.87±0.06	1.38±0.08	1.79±0.10	<0.001
Whole grains	10	1.91±0.09	1.53±0.11	2.18±0.14	2.13±0.15	<0.001
Dairy	10	3.58±0.08	3.18±0.12	3.74±0.14	4.00±0.15	<0.001
Total protein foods	5	4.21±0.03	4.05±0.05	4.31±0.05	4.32±0.05	<0.001
Seafood and plant proteins	5	1.73±0.05	1.30±0.07	1.77±0.08	2.34±0.10	<0.001
Fatty acids	10	5.09±0.08	4.84±0.13	5.26±0.13	5.26±0.16	<0.001
Sodium	10	4.96±0.09	5.26±0.14	5.06±0.14	4.40±0.16	0.028
Refined grains	10	6.25±0.08	5.94±0.13	6.37±0.14	6.58±0.15	<0.001
Empty calories	20	8.86±0.16	7.62±0.24	9.22±0.26	10.27±0.31	0.001
						

^a^ P-value for trend across tertiles of MVD within each sex group

^b^ 2-sided P-value from independent sample *t*-test comparing means of HEI-2010 total scores and components across sex groups. Ranges and means within each tertile of MVD are presented in Fig 1 for both sexes combined.

Whole fruit was among the components that exhibited more than a doubling effect when going from T_1_ to T_3_ in both men and women (0.61 to 1.38 among men; 0.87 to 1.79 among women). In contrast, components such as empty calories, refined grains, total protein foods, whole grains, total fruits, greens and beans, total vegetables, dairy (among women) and fatty acids (among women) exhibited a linear trend with a mean relative increase of 31.4%. The range of relative increase in means between T_1_ and T_3_ of MVD was between 5% (total protein foods in men) to 100% (Greens and beans in women). Among women only, higher diet cost was associated with a lower score on the HEI-2010 sodium component.

Overall, men had lower HEI-2010 score compared to women, with lower scores also observed in men on 6 components including total vegetables, greens and beans, total fruit, whole fruit, whole grains, seafood and plant proteins and the reverse pattern for total proteins (men>women). Yet the component scores for greens and beans, total and whole fruit were ≤ 2 out of 5 and whole grains were <4 out of 10, reflecting only 40% compliance with guidelines and indicating major improvements in food selections are needed. Similar results were observed in women.

**Table 3** presents bivariate associations between the measures of nutrient adequacy and MVD tertiles. A linear and positive dose-response relationship was found for both sexes between diet cost and all 16 NARs, as well as the MAR. Overall, men had higher MAR compared to women (unlike 2010-HEI), and this pattern was observed for most NAR, with few exceptions. In contrast to the HEI-2010 scores, improvements in NAR scores over MVD tertiles indicated either achieving or exceeding 67% which would suggest adequate nutrient intake.

**Table 3:** Mean and Nutrient Adequacy Ratio (MAR and NAR) Scores^a^ for HANDLS participants by monetary value of diet tertile and sex.

		**Men (n = 918)**			
		**MVD tertiles ($/d)**			
	All	**T1**	**T2**	**T3**	P-trend ^b^
		**0.9–4.1**	**>4.1–6.0**	**>6.0–31.0**	
	**X±SEM**	**X±SEM**	**X±SEM**	**X±SEM**	
MAR	76.8±0.5 ^c^	62.7±0.9	76.8±0.6	85.2±0.4	<0.001
Vitamin A, NAR	56.3±1.0 ^c^	39.5±1.7	54.0±1.7	68.1±1.4	<0.001
Vitamin C, NAR	53.9±1.2	32.7±2.0	55.5±2.1	65.4±1.7	<0.001
Vitamin D, NAR	27.7±0.8^c^	17.0±0.9	26.7±1.2	34.9±1.3	<0.001
Vitamin E, NAR	45.1±0.8^c^	27.1±1.0	40.9±1.1	59.1±1.2	<0.001
Vitamin B-6, NAR	88.9±0.7^c^	72.2±1.5	90.4±0.9	97.8±0.4	<0.001
Vitamin B-12, NAR	94.4±0.5^c^	85.5±1.6	96.2±0.7	98.4±0.4	<0.001
Thiamin, NAR	90.1±0.6^c^	76.7±1.5	91.9±0.8	96.8±0.5	<0.001
Riboflavin, NAR	95.0±0.4 ^c^	86.8±1.3	96.5±0.6	98.7±0.3	<0.001
Niacin, NAR	95.4±0.4^c^	87.4±1.1	96.7±0.6	99.4±0.2	<0.001
Folate, NAR	77.6±0.8^c^	58.0±1.4	77.3±1.2	89.7±0.9	<0.001
Iron, NAR	97.5±0.3^c^	92.5±0.9	98.9±0.4	99.4±0.2	<0.001
Copper, NAR	93.2±0.5^c^	79.5±1.2	95.7±0.6	99.6±0.1	<0.001
Zinc, NAR	85.0±0.6	67.3±1.5	86.4±1.0	94.5±0.7	<0.001
Calcium, NAR	70.1±0.9^c^	49.0±1.4	68.1±1.3	84.1±1.1	<0.001
Magnesium, NAR	60.8±0.8^c^	39.0±0.9	55.3±0.9	78.1±0.9	<0.001
Phosphorus, NAR	97.5±0.3^c^	92.2±1.0	98.8±0.4	99.8±0.1	<0.001
		**Women (n = 1193)**			
		**MVD tertiles ($/d)**			
	All	**T1**	**T2**	**T3**	P-trend ^b^
		**0.9–4.1**	**>4.1–6.0**	**>6.0–31.0**	
	X±SEM	X±SEM	X±SEM	X±SEM	
MAR	69.9±0.5	58.3±0.7	73.6±0.6	82.6±0.6	<0.001
Vitamin A, NAR	50.1±0.9	36.7±1.2	52.9±1.3	66.5±1.6	<0.001
Vitamin C, NAR	51.4±1.0	37.6±1.5	54.1±1.6	68.4±1.8	<0.001
Vitamin D, NAR	21.2±0.6	13.8±0.6	22.1±0.9	31.2±1.4	<0.001
Vitamin E, NAR	38.8±0.7	27.1±0.8	39.8±1.0	55.1±1.4	<0.001
Vitamin B-6, NAR	82.9±0.6	70.8±1.1	88.2±0.9	94.0±0.8	<0.001
Vitamin B-12, NAR	88.7±0.6	79.4±1.2	93.3±0.8	96.6±0.7	<0.001
Thiamin, NAR	85.3±0.6	75.2±1.1	89.7±0.8	94.6±0.7	<0.001
Riboflavin, NAR	92.8±0.4	86.2±0.9	96.6±0.5	97.7±0.5	<0.001
Niacin, NAR	88.6±0.5	79.8±1.0	92.9±0.7	96.1±0.6	<0.001
Folate, NAR	68.6±0.7	55.0±1.1	71.7±1.1	84.8±1.2	<0.001
Iron, NAR	64.7±0.7	51.2±1.0	67.8±1.0	80.9±1.2	<0.001
Copper, NAR	87.5±0.5	74.2±1.0	93.8±0.5	99.3±0.2	<0.001
Zinc, NAR	85.2±0.6	73.1±1.1	91.0±0.8	95.7±0.7	<0.001
Calcium, NAR	56.2±0.8	40.1±0.9	58.6±1.1	77.4±1.2	<0.001
Magnesium, NAR	63.7±0.7	46.6±0.8	67.6±0.9	84.5±1.0	<0.001
Phosphorus, NAR	93.1±0.4	85.4±0.9	97.8±0.4	98.5±0.4	<0.001

^a^ All scores (MAR and NAR) ranged between 0 and 100.

^b^ P-value for trend across tertiles of MVD within each sex group

^c^ 2-sided P-value from independent sample *t*-test comparing means of NAR and MAR across sex groups.

Racial and poverty status disparities were also found for both 2010-HEI and MAR scores, as shown in **S2 Table**. Specifically, the total mean HEI-2010 score was higher for people above poverty compared to below poverty, notably for total vegetables, total and whole fruit components, dairy, whole grains, seafood and plant proteins, and empty calories. Racial differences were inconsistent for 2010-HEI components and were not detected for the total HEI-2010 score. In contrast, a higher mean MAR total score was found among Whites compared African-Americans. Whites had higher NAR than African-American for most nutrients, with the exception of vitamin C (African-Americans > Whites), niacin, iron and copper (no difference). Additionally, Vitamins C and E, calcium and magnesium NAR were higher among individuals above poverty compared to those below poverty.

### HEI-2010 and components: adjusted association with MVD, stratified analysis by sex, race and poverty status

**Table 4** displays associations between MVD expressed as $/day and HEI-2010 (total score and component scores) based on multiple regression models that adjusted for sex, race, poverty status, age, education, literacy, unemployment status, current smoking status, % energy from grocery stores, self-rated health and daily energy intake. Multiple OLS models indicated that a $1/day higher MVD was associated with a 1.65±0.12 higher HEI-2010 total score overall, with significant differences by sex, race and poverty status. Given that the interquartile range IQR for MVD was $3.70/d (Q1) to $6.62/d (Q4), the results then suggest that a $3/day higher MVD was associated with a 4.98±0.35 higher total HEI-2010. A stronger magnitude of the association was noted among Whites compared to African Americans and among participants above poverty compared to below poverty.

**Table 4:** Association between monetary value of food ($/day) and HEI-2010 (and components): findings from multiple OLS (total HEI-2010 score) and logistic regression models (components: upper tertile vs. two lower tertiles combined (referent category))^a^.

	ALL		Men		Women		Whites		African-Americans		Above poverty		Below poverty	
	(n = 2109)		(n = 917)		(n = 1,192)		(n = 893)		(n = 1,216)		(n = 1,204)		(n = 905)	
***OLS regression*, *β±SE***														
2010-HEI, per1$/day	+1.65	±0.12^§^	+1.43	±0.15^§^	+1.92	±0.18^§^ ^,^ ^b^	+1.92	±0.19^§^	+1.38	±0.15^§^ ^,^ ^c^	+2.03	±0.16^§^	+1.18	±0.16^§^ ^,^ ^d^
2010-HEI, per 3$/day	+4.98	±0.35^§^	__		__		__							
***Multiple logistic regression*, *OR(95%CI)***														
Total vegetables(≥3.3 vs. <3.3)	1.24	(1.18;1.31) ^§^	1.24	(1.15;1.33) ^§^	1.28	(1.19;1.39) ^§^	1.24	(1.15;1.34) ^§^	1.23	(1.15;1.32) ^§^ ^,^ ^c^	1.23	(1.15;1.32) ^§^	1.24	(1.16;1.35) ^§^
Greens and beans(≥2.5 vs. <2.5)	1.25	(1.19,1.31) ^§^	1.22	(1.14;1.31) ^§^	1.27	(1.18;1.38) ^§^	1.29	(1.19;1.40) ^§^	1.22	(1.15;1.31) ^§^	1.25	(1.17;1.34) ^§^	1.24	(1.15;1.34) ^§^
Total fruit(≥2.5 vs. <2.5)	1.29	(1.18;1.29) ^§^	1.12	(1.05;1.20)*	1.21	(1.12;1.30) ^§^	1.20	(1.10;1.31) ^§^	1.14	(1.07;1.22) ^§^	1.17	(1.09;1.25) ^§^	1.15	(1.07;1.25) ^§^
Whole fruit(≥1.6 vs. <1.6)	1.23	(1.17;1.30) ^§^	1.21	(1.12;1.29) ^§^	1.26	(1.17;1.36) ^§^	1.26	(1.15;1.36) ^§^	1.21	(1.13;1.29) ^§^	1.24	(1.17;1.33) ^§^	1.20	(1.11;1.30) ^§^
Whole grains(≥1.8 vs. <1.8)	1.06	(1.01;1.11)*	1.03	(0.97;1.10)	1.09	(1.01;1.17)*	1.06	(0.98;1.14)	1.01	(0.95;1.08) ^c^	1.07	(1.00;1.16)*	1.03	(0.95;1.11)
Dairy(≥4.7 vs. <4.7)	1.03	(0.99;1.09)	1.02	(0.95;1.10)	1.06	(1.00;1.14) ^b^	1.21	(1.12;1.31) ^§^	1.02	(0.96;1.10)	1.10	(1.03;1.18)*	0.98	(0.91;1.06)
Total protein foods(≥4.4 vs. <4.4)	1.23	(1.16;1.29)*	1.24	(1.15;1.34) ^§^	1.22	(1.13;1.32) ^§^	1.22	(1.13;1.33) ^§^	1.25	(1.16;1.35) ^§^	1.23	(1.14;1.32) ^§^	1.23	(1.13;1.35) ^§^
Seafood and plant proteins(≥2.5 vs. <2.5)	1.20	(1.14;1.26) ^§^	1.18	(1.10;1.27) ^§^	1.23	(1.16;1.32) ^§^	1.07	(0.98;1.16)	1.19	(1.11;1.27) ^§^	1.26	(1.17;1.35) ^§^	1.14	(1.05;1.23)* ^,^ ^d^
Sodium(≥6.4 vs. <6.4)	0.83	(0.79;0.88) ^§^	0.87	(0.81;0.94) ^§^	0.79	(0.73;0.86) ^§^ ^,^ ^b^	0.84	(0.77;0.91) ^§^	0.83	(0.78;0.90) ^§^	0.82	(0.76;0.88) ^§^	0.85	(0.79;0.92) ^§^
Refined grains(≥7.8 vs. <7.8)	1.10	(1.05;1.16) ^§^	1.04	(1.01;1.16)*	1.13	(1.06;1.22) ^§^	1.12	(1.04;1.22)*	1.10	(1.03;1.17) *	1.12	(1.05;1.20)*	1.09	(1.02;1.17)*
Empty calories(≥6.0 vs. <6.0)	1.36	(1.29;1.44) ^§^	1.31	(1.21;1.41) ^§^	1.43	(1.32;1.55) ^§^ ^,^ ^b^	1.36	(1.24;1.47) ^§^	1.36	(1.27;1.47) ^§^	1.38	(1.28;1.48) ^§^	1.34	(1.24;1.46) ^§^

*P<0.05

^§^P<0.001

^a^ Models were adjusted for energy intake (kcal/d), sex, race, poverty status (whenever not used for stratification), age, education (<HS, HS, >HS; 2 dummy variables), literacy (WRAT-3 total score); unemployment status (0 = no, 1 = yes, 9 = missing; 2 dummy variables), current smoking status (0 = never or former, 1 = current smoker, 9 = missing, 2 dummy variables), current drug use (0 = never or >6 months ago; 1 = <6 months ago; 9 = missing; 2 dummy variables), % energy obtained at grocery stores, self-rated health (1 = poor, 2 = fair, 3 = good, 4 = fair, 5 = excellent: four dummy variables).

We additionally examined the MVD-Fatty acid relationship and found that it was not statistically significant.

^b^ p<0.10 for null hypothesis that interaction term between sex and food cost is = 0. Interaction terms are added to the full non-stratified model with main effect of sex and food cost as well as other covariates.

^c^ p<0.10 for null hypothesis that interaction term between race and food cost is = 0. Interaction terms are added to the full non-stratified model with main effect of race and monetary value of diet as well as other covariates.

^d^ p<0.10 for null hypothesis that interaction term between poverty status and monetary value of diet is = 0. Interaction terms are added to the full non-stratified model with main effect of poverty status and monetary value of diet as well as other covariates.

Based on multiple logistic regression models with outcome being uppermost tertile vs. lower two tertiles combined for each HEI-2010 component, in the total population, a higher odds of better dietary quality was found with greater MVD for only 9 of the 12 components. These components were total vegetables, greens and beans, total fruits, whole fruits, whole grains, total protein foods, seafood and plant proteins, refined grains and empty calories. In contrast, and as was shown in the bivariate analysis, a greater MVD was linked to a lower likelihood of a better score on the HEI-2010 sodium component.

Stratified models by sex, race and poverty status indicated a few differences in the association between MVD and likelihood of better dietary quality on each HEI-2010 component (Table 4). Most notably, women were more likely than men to score higher on the “empty calories” components with greater MVD, while participants above poverty were more likely to score high on the “seafood and plant protein” component than below poverty participants.

**Fig 2:**
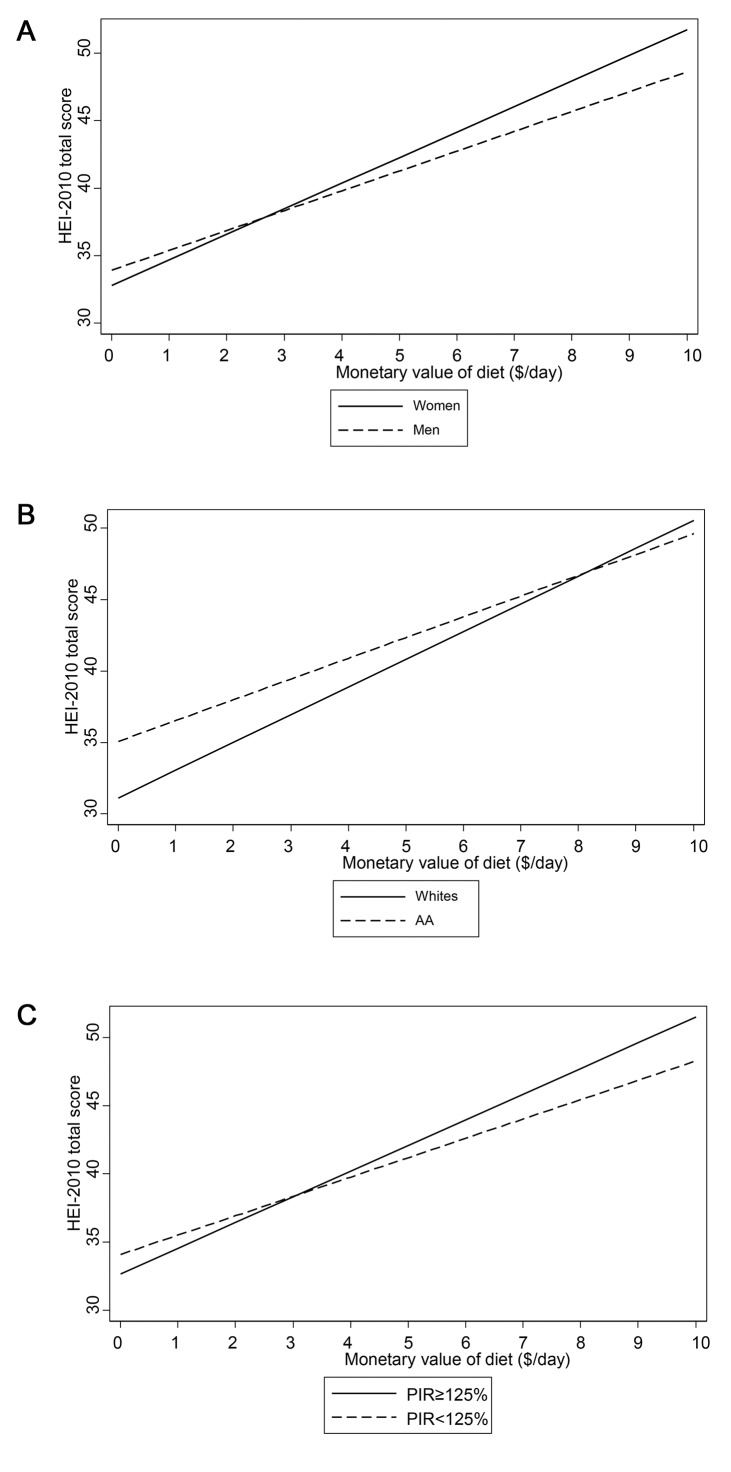
A-C. Predicted mean HEI-2010 total score by monetary value of diet (MVD, $/day), stratified by sex, race and poverty status from multiple OLS model with interaction between MVD and sex^a^, race^b^, and poverty status^c^ HANDLS study, wave 1. ^a^p<0.10 for MVD×sex interaction; ^b^p<0.10 for MVD×race interaction; ^c^p<0.10 for MVD×pov interaction

**Fig 2A–2C** illustrate the finding of an interaction between MVD and each of the three main socio-demographic variables of interest, namely sex, race and poverty status, in its relationship with HEI-2010 total score, using multiple OLS regression model. Based on those figures, stronger associations were found among women (**Fig 2A**), Whites (**Fig 2B**) and participants above poverty (**Fig 2C**.

### Nutrient adequacy: adjusted association with monetary value of diet, stratified analysis by sex, race and poverty status

**Table 5** displays similar associations between MVD ($/day) and nutrient adequacy (MAR and NAR) based on multiple regression models that adjusted for the same factors as in **Table 4**. Multiple OLS models indicated that a $1/day higher MVD was associated with a 1.29±0.12 higher MAR total score overall (i.e. a 3.88±0.37 higher MAR score with each $3/day higher MVD), with significant differences by sex and poverty status, and stronger effects among women and ≥125%PIR. Similar patterns of differential associations by sex were found for most NAR components, with the exception of vitamin D. Similarly, significant differentials by poverty status (p<0.10 for interaction between MVD and poverty status), with stronger associations found among participants above poverty in the case of all micronutrients with the possible exception of vitamins A, C and D. No detectable differences by race for the MAR-MVD association was found and the differences for component NAR by race were inconsistent.

**Table 5:** Association between monetary value of reported diets ($/day) and mean adequacy ratio (MAR) and component nutrient adequacy ratios (NAR): findings from multiple OLS regression models^a^.

	ALL		Men		Women		Whites		African-Americans		Above poverty		Below poverty	
	(n = 2109)		(n = 917)		(n = 1,192)		(n = 893)		(n = 1,216)		(n = 1,204)		(n = 905)	
***OLS regression*, *β±SE***														
MAR	1.29	±0.12^§^	1.02	±0.15^§^	1.73	±0.18^§^ ^,^ ^b^	1.22^§^	±0.20^§^	1.25	±0.16^§^	1.68	±0.16^§^	0.83	±0.19^§^ ^,^ ^d^
MAR, per 3$/day	3.88	±0.37^§^	__		__		__		__		__		__	
Vitamin A, NAR	2.17	±0.30^§^	1.91	±0.41^§^	2.59	±0.43^§^ ^,^ ^b^	1.87	±0.46^§^	2.24	±0.39^§^	2.08	±0.40^§^	2.10	±0.45^§^
Vitamin C, NAR	3.37	±0.37^§^	2.90	±0.52^§^	3.98	±0.52^§^ ^,^ ^b^	3.62	±0.55^§^	3.06	±0.50^§^	3.67	±0.50^§^	2.85	±0.55^§^
Vitamin D, NAR	1.26	±0.22^§^	1.42	±0.33^§^	1.11	±0.30^§^ ^,^ ^b^	0.89	±0.36*	1.53	±0.28^§^	1.52	±0.20^§^	1.00	±0.33^§^
Vitamin E, NAR	1.69	±0.20^§^	1.41	±0.28^§^	2.09	±0.30^§^ ^,^ ^b^	2.20	±0.33^§^	1.24	±0.26^§^ ^,^ ^c^	2.43	±0.28^§^	0.86	±0.30* ^,^ ^d^
Vitamin B-6, NAR	1.17	±0.20^§^	0.97	±0.25^§^	1.52	±0.31^§^ ^,^ ^b^	1.08	±0.33^§^	1.09	±0.26^§^	1.77	±0.27^§^	0.50	±0.31^,^ ^d^
Vitamin B-12,NAR	0.77	±0.21^§^	0.46	±0.23*	1.20	±0.33^§^ ^,^ ^b^	0.48	±0.30	0.90	±0.28*	0.98	±0.29*	0.59	±0.30* ^,^ ^d^
Thiamin, NAR	0.18	±0.19	0.15	±0.23	0.30	±0.29 ^b^	-0.04	±0.28	0.28	±0.26 ^c^	0.40	±0.25	-0.07	±0.30^,^ ^d^
Riboflavin, NAR	0.08	±0.15	-0.08	±0.19	0.30	±0.23 ^b^	-0.07	±0.21	0.14	±0.21 ^c^	0.39	±0.20	-0.28	±0.22^,^ ^d^
Niacin, NAR	0.28	±0.17	+0.22	±0.18	0.52	±0.26* ^,^ ^b^	0.27	±0.27	0.21	±0.21	0.63	±0.22*	-0.13	±0.25^,^ ^d^
Folate, NAR	1.08	±0.23^§^	+0.84	±0.29^§^	1.51	±0.34^§^ ^,^ ^b^	1.21	±0.36*	0.94	±0.30	1.65	±0.30^§^	-0.37	±0.35^,^ ^d^
Iron, NAR	0.29	±0.20	-0.03	±0.13	1.09	±0.31^§^ ^,^ ^b^	0.27	±0.32	0.17	±0.25	0.63	±0.26*	-0.11	±0.29^,^ ^d^
Copper, NAR	1.74	±0.16^§^	+1.13	±0.19^§^	2.57	±0.24^§^ ^,^ ^b^	1.49	±0.25^§^	1.82	±0.21^§^	2.19	±0.21^§^	1.14	±0.25^§^ ^,^ ^d^
Zinc, NAR	0.96	±0.20^§^	+0.76	±0.27^§^	1.26	±0.30^§^ ^,^ ^b^	0.84	±0.31^§^	0.88	±0.26^§^	1.63	±0.27^§^	0.22	±0.30^,^ ^d^
Calcium, NAR	1.46	±0.23^§^	1.68	±0.32^§^	3.54	±0.32^§^ ^,^ ^b^	2.19	±0.37^§^	2.56	±0.29^§^	2.89	±0.30^§^	1.99	±0.35^§^ ^,^ ^d^
Magnesium, NAR	2.96	±0.17^§^	2.58	±0.22^§^	3.51	±0.29^§^ ^,^ ^b^	3.26	±0.29^§^	2.59	±0.21^§^	3.59	±0.22^§^	2.19	±0.26^§^ ^,^ ^d^
Phosphorus, NAR	0.23	±0.13	0.04	±0.13	0.57	±0.21* ^,^ ^b^	-0.05	±0.19	0.40	±0.18* ^,^ ^c^	0.41	±0.18*	-0.02	±0.19^,^ ^d^

*P<0.05

^§^P<0.001

^a^ Models were further adjusted for energy intake (kcal/d), sex, race, poverty status (whenever not used for stratification), age, education (<HS, HS, >HS; 2 dummy variables), literacy (WRAT-3 total score); unemployment status (0 = no, 1 = yes, 9 = missing; 2 dummy variables), current smoking status (0 = never or former, 1 = current smoker, 9 = missing, 2 dummy variables), current drug use (0 = never or >6 months ago; 1 = <6 months ago; 9 = missing; 2 dummy variables), % energy obtained at grocery stores, self-rated health (1 = poor, 2 = fair, 3 = good, 4 = fair, 5 = excellent: four dummy variables).

^b^ p<0.10 for null hypothesis that interaction term between sex and monetary value of diet is = 0. Interaction terms are added to the full non-stratified model with main effect of sex and monetary value of diet as well as other covariates.

^c^ p<0.10 for null hypothesis that interaction term between race and monetary value of diet is = 0. Interaction terms are added to the full non-stratified model with main effect of race and monetary value of diet as well as other covariates.

^d^p<0.10 for null hypothesis that interaction term between poverty status and monetary value of diet is = 0. Interaction terms are added to the full non-stratified model with main effect of poverty status and monetary value of diet as well as other covariates.

**Fig 3:**
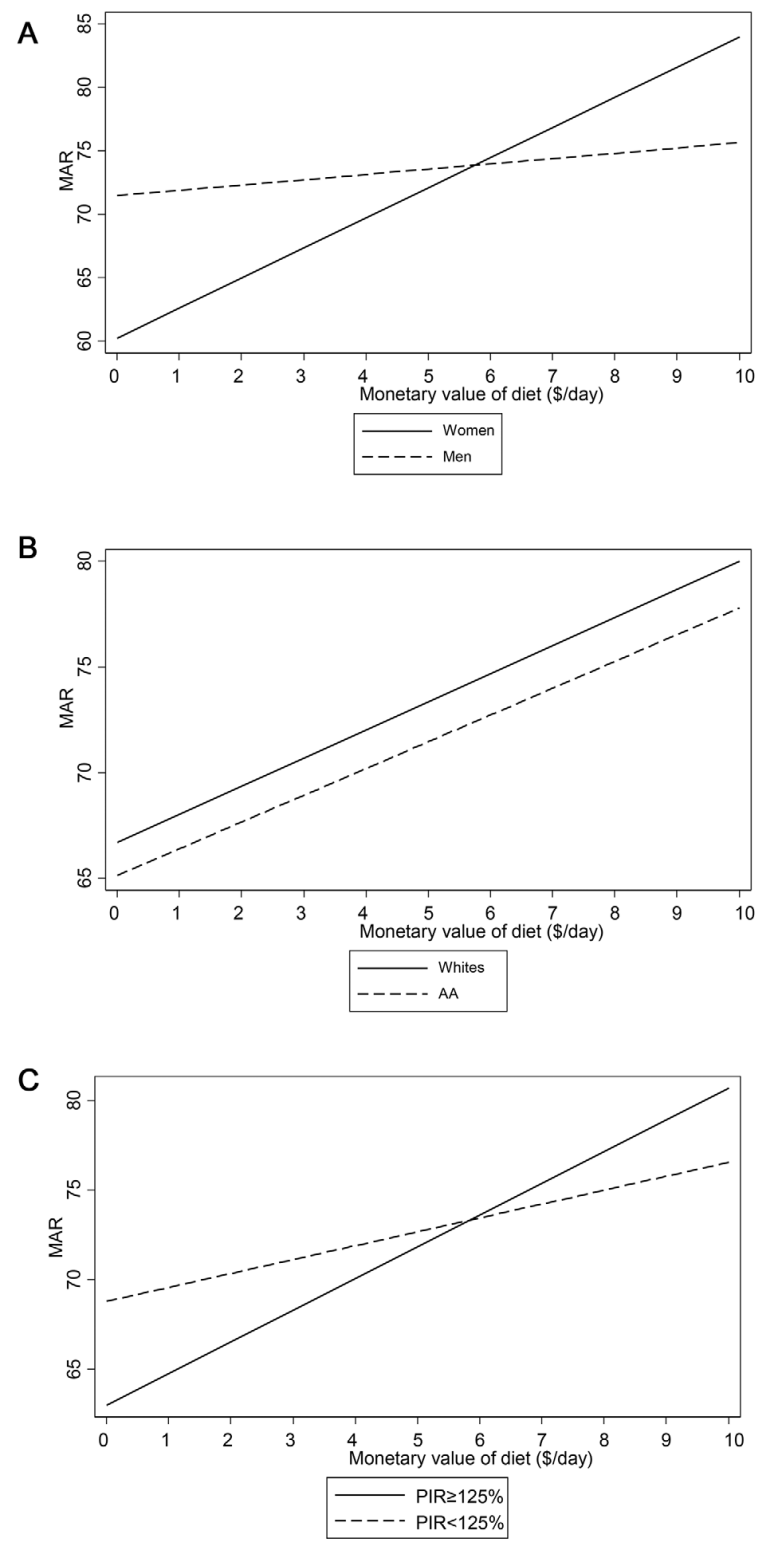
A-C. Predicted mean MAR by monetary value of diet (MVD, $/day), stratified by sex, race and poverty status, from multiple OLS model with interaction between MVD and sex^a^, race^b^, and poverty status^c^; HANDLS study, wave 1. ^a^p<0.10 for MVD×sex interaction; ^b^p<0.10 for MVD×race interaction; ^c^p<0.10 for MVD×pov interaction.

**Fig 3A–3C** illustrate the finding of an interaction between MVD and each of the three main socio-demographic variables of interest, namely sex, race and poverty status, in its relationship with the MAR score, using multiple OLS regression model. Based on those figures, stronger associations were found among women (**Fig 3A**) and participants above poverty (**Fig 3C**), with no significant difference in the association by race (**Fig 3B**).

## Discussion

This research is the first to link MVD to dietary quality using the HEI-2010 and MAR scores simultaneously among urban US adults, while comprehensively examining differential associations across sex, race and poverty status. For both diet quality indices, stronger associations were observed among participants above poverty, whereas for HEI-2010 stronger associations were noted also among Whites and for MAR among women.

In our study, even the uppermost MVD tertile was associated with an HEI-2010 score less than 50 out of 100 in both men and women, indicating many improvements in food choices are needed to improve diet quality. The NAR scores, unadjusted for energy, increased over the tertiles but the scores for Vitamins A, C, E and D, magnesium, and calcium (for women only) of the highest tertile were still less than 70 out of 100, suggesting inadequacy. These nutrients are provided by fruits, vegetables and dairy products. The results provide evidence that a higher odds of better diet quality was found with greater MVD for the fruit and vegetable components of the HEI-2010.

Since the CNPP food price database reflects all foods purchased in retail settings and prepared at home, estimated actual food expenditures will exceed the MVD. Importantly, a $3/day higher MVD was associated with a 4.98±0.35 higher total HEI-2010 and a 3.88±0.37 higher MAR score.

Other researchers corroborate our findings. Bernstein and colleagues reported that energy-adjusted MVD (uppermost vs. lowest quintile) was associated with a 30 point higher level of the Alternative Healthy Eating Index score for women enrolled in the US Nurses’ Health Study, N = 78,191.[34] Aggarwal and colleagues found that MVD mediated the pathway between income and diet quality measures, which was further moderated by education.[39] Using the same sample of participants (N = 1,266), they also showed that MVD were positively related to higher intakes of dietary fiber, Vitamins A, C, D, E, and B-12, β-carotene, folate, iron, calcium, potassium, and magnesium. In contrast, lower MVD were linked to higher intakes of saturated fats, trans fats, and added sugars.[43] In a study of French adults, participants in the highest MAR tertile had the lowest dietary energy density and the highest diet costs.[27] A similar finding was reported by at least one other cross-sectional study that used MAR as a measure of dietary quality.[39]

Using data from 2001–2002 NHANES and a similar analytical approach with the HEI-2005, Rehm and colleagues also found that MVD were significantly associated with overall diet quality. They also reported that higher MVD was strongly associated with consuming more servings of fruit and vegetables and fewer calories from solid fat, alcoholic beverages, and added sugars.[41] However, their analysis did not adjust for major confounders and used energy-adjusted diet costs ($/kcal) as main predictors rather than the actual cost ($/day). Using the HEI and Mediterranean Diet Score, another study drew similar conclusions about the association of MVD with healthier diets among adults.[70] Using a more recent waves of NHANES (2007–2010) and the 2010-HEI, Rehm and colleagues also demonstrated that disparities dietary quality among US adults were largely determined by food prices and by energy-adjusted diet costs. In particular, the study found that lower diet costs were associated with lower consumption of vegetables, fruits, whole grains, and seafood, and higher consumption of refined grains and solid fat, alcohol and added sugars. The relationship between energy-adjusted diet cost and overall diet quality was stronger among women compared to men (similar to our finding) with no racial differences detected (at odds with our findings). [51]

Morever, in our study, the sodium moderation component of the HEI-2010 which was inversely associated with diet cost even after adjustment for energy intake. This was comparable to another study whereby MVD was positively associated with biomarker-based estimates of protein, potassium and sodium intake in young Japanese women. These associations appeared mainly to be explained by the monetary costs of vegetables and fish.[32]

Using one of the largest cross-sectional data sets available among women in the United Kingdom (N = 35,000), Morris and colleagues showed that dietary diversity, in addition to a healthful diet pattern, was associated with increased MVD. Moreover, women with higher SES in terms of education and occupation were more likely to consume a more healthful diet.(44) Individual studies also indicated that MVD vs. dietary quality associations were stronger among US women compared to men. [30, 31, 41]

The study findings provide evidence that reported intakes which are more compliant with food- and nutrient-based recommendations are associated with greater monetary value of the diet. A recent meta-analysis of 27 studies from 10 countries indicated that, among food groups price differences existed and that healthier diets cost more than the least healthful diets. However, price differences were not found significant for nutrient-based patterns.[71]

Among its strengths, this study is the first to utilize the HEI-2010 and MAR scores to test the association between the monetary cost of reported diets and overall dietary quality. Furthermore while examining differentials in diet quality measured with two alternate indices, adjustments were made for key confounders; specifically race, sex and income. The findings contribute to our knowledge about an understudied urban African American and White population which is representative of at least 14 urban settings across the United States.

However, the results of our study have to be interpreted with caution in light of some key limitations. Among them is our inability to utilize time-varying price data unique to Baltimore residences that accounted for seasonality of the prices, resulting in an assumption that overall food price structure for Baltimore residents from the entire sample are those faced by Americans nationally in 2003–2004. To date, no study has tested this assumption thoroughly even though applying national price indices is the key method that other cited studies have used.(39) However, A recent study suggests that using food prices to estimate MVD can give an accurate though underestimated picture of actual food expenditures. [12] Moreover, in an earlier era, extensive research suggested that the poor with their limited to lower volume purchases and reduced ability to travel, paid more for their food which indicates our results, if anything, underestimate the income differentials in diet quality.[72, 73] An example of this is one unpublished study using Homescan data found that low income blacks are less likely to shop at Walmart than other low and middle income Americans.[74]

In conclusion, the association between MVD and dietary quality based on HEI-2010 and MAR scores was positive overall. Sex, race, and poverty status modulated our findings. For instance, stronger associations were found for participants above poverty for both diet quality measures. Despite this differential positive association, approaching compliance with the Dietary Guidelines (at least 80 out of 100 for HEI-2010) requires a substantially higher MVD. Consequently, nutrition education may further improve people’s decision-making regarding food venues and dietary choices. For the HANDLS study population, it is thus apparent that both more selective food choices and money are needed to improve diet quality. Therefore, higher income may be the key driving force for the effectiveness of higher MVD leading to better dietary quality. This study is observational, but given the longitudinal design of the HANDLS study the relationship between MVD and dietary quality can be examined in the future. The findings also suggest that future intervention studies should be conducted on urban population like ours and differential effects of increasing MVD on dietary quality should be studied carefully taking into account sex, race and poverty status.

## Supporting Information

S1 Fig Mean HEI component scores; HANDLS study, wave 1. (PPTX)

S2 Fig Mean MAR and NAR scores; HANDLS study, wave 1. (PPTX)

S1 Table Baseline characteristics and MVD distribution of selected HANDLS sample by sex, race and poverty status groups; HANDLS 2003–2009. (DOCX)

S2 Table 2010-Healthy Eating Index and MAR/NAR scores by race and poverty status groups; HANDLS 2003–2009. (DOCX)

## Abbreviations

2010-HEI: Healthy Eating Index, 2010 version
FNDDS: Food and Nutrient Database for Dietary Studies
HANDLS: Healthy Aging in Neighborhood of Diversity across the Lifespan
HS: High School
MAR: Mean Adequacy Ratio
MVD: Monetary value of the diet
NAR: Nutrient Adequacy Ratio
NHANES: National Health and Nutrition Examination Surveys
PIR: Poverty Income Ratio
RDA: Recommended Dietary Allowance
SE: Standard Error
USDA: US Department of Agriculture
