# Supplementary material for: Monetary Value of Diet Is Associated with Dietary Quality and Nutrient Adequacy among Urban Adults, Differentially by Sex, Race and Poverty Status
Source: PLoS One. 2015 Nov 4;10(11):e0140905. doi: 10.1371/journal.pone.0140905 (PMC4633204; doi:10.1371/journal.pone.0140905)
Supplement: S1 Fig — (PPTX) [file pone.0140905.s001.pptx]

## Slide 1
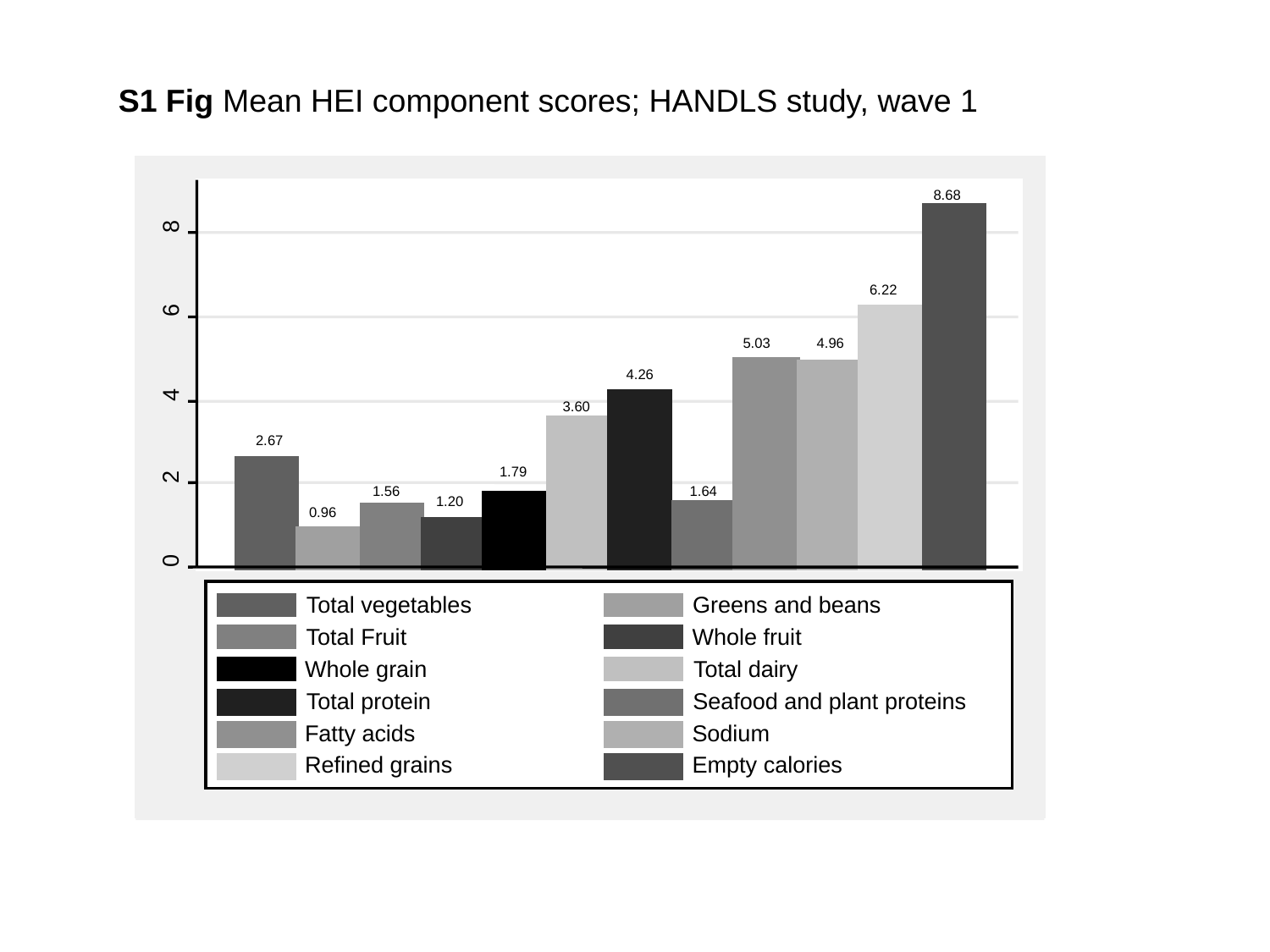

S1 Fig Mean HEI component scores; HANDLS study, wave 1
8
6
4
2
0
Total vegetables
Greens and beans
Total Fruit
Whole fruit
Whole grain
Total dairy
Total protein
Seafood and plant proteins
Fatty acids
Sodium
Refined grains
Empty calories
8.68
6.22
5.03
4.96
4.26
3.60
2.67
1.79
1.56
1.64
1.20
0.96
