# Supplementary material for: Monetary Value of Diet Is Associated with Dietary Quality and Nutrient Adequacy among Urban Adults, Differentially by Sex, Race and Poverty Status
Source: PLoS One. 2015 Nov 4;10(11):e0140905. doi: 10.1371/journal.pone.0140905 (PMC4633204; doi:10.1371/journal.pone.0140905)
Supplement: S2 Fig — (PPTX) [file pone.0140905.s002.pptx]

## Slide 1
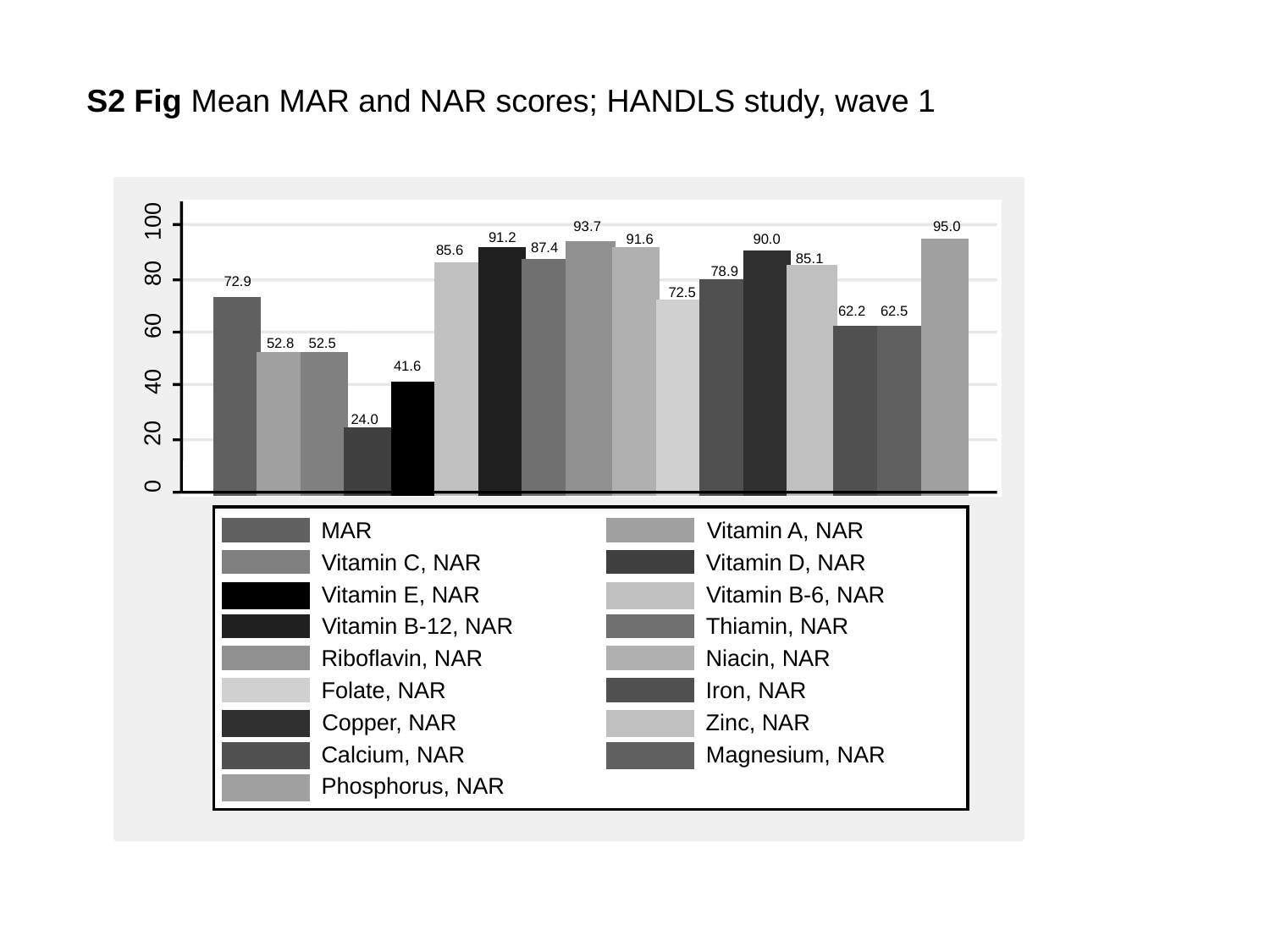

S2 Fig Mean MAR and NAR scores; HANDLS study, wave 1
100
80
60
40
20
0
MAR
Vitamin A, NAR
Vitamin C, NAR
Vitamin D, NAR
Vitamin E, NAR
Vitamin B-6, NAR
Vitamin B-12, NAR
Thiamin, NAR
Riboflavin, NAR
Niacin, NAR
Folate, NAR
Iron, NAR
Copper, NAR
Zinc, NAR
Calcium, NAR
Magnesium, NAR
Phosphorus, NAR
93.7
95.0
91.2
91.6
90.0
87.4
85.6
85.1
78.9
72.9
72.5
62.2
62.5
52.8
52.5
41.6
24.0
