# Supplementary material for: Monetary Value of Diet Is Associated with Dietary Quality and Nutrient Adequacy among Urban Adults, Differentially by Sex, Race and Poverty Status
Source: PLoS One. 2015 Nov 4;10(11):e0140905. doi: 10.1371/journal.pone.0140905 (PMC4633204; doi:10.1371/journal.pone.0140905)
Supplement: S1 Table — (DOCX) [file pone.0140905.s003.docx]

**S1 Table.** Baseline characteristics and MVD distribution of selected HANDLS sample by sex, race and poverty status groups; HANDLS 2003-2009

|  | **Men** | **Women** | **Whites** | **African-Americans** | **Above Poverty** | **Below poverty** |
| --- | --- | --- | --- | --- | --- | --- |
| **Monetary value of diet and energy intakes** |  |  |  |  |  |  |
| Monetary value of diet, $/day  (X ± SE) | 6.2±0.1 | 5.0±0.1^a^ | 5.6±0.1 | 5.4±0.1 | 5.6±0.1 | 5.4±0.1 |
| Energy intake, kcal/d (X ± SE) | 2363±36 | 1,743±22 ^a^ | 2028±31 | 2001±29 | 1982±26 | 2053±35 |
|  |  |  |  |  |  |  |
| **Socio-demographic and SES variables** |  |  |  |  |  |  |
| Sex, % male | __ | __ | 42.9 | 43.9 | 46.1 | 40.1^a^ |
|  |  |  |  |  |  |  |
| Age, yrs. (X ± SEM) | 48.4±0.3 | 48.4±0.3 | 48.5±0.3 | 48.3±0.3 | 48.7±0.3 | 48.0±0.3 |
|  |  |  |  |  |  |  |
| African-American, % | 43.9 | 43.0 | __ | __ | 49.3 | 68.9^a^ |
|  |  |  |  |  |  |  |
| Income, % (<125% PIR) | 40.1 | 46.1^a^ | 31.6 | 51.3^a^ | __ | __ |
|  |  |  |  |  |  |  |
| Education, yrs. completed (X ± SE) | 12.4±0.1 | 12.4±0.1 | 12.8±0.1 | 12.2±0.1 ^a^ | 13.2±0.1 | 11.6±0.1^a^ |
| <HS | 7.6 | 6.0 | 9.3 | 4.8 ^a^ | 4.7 | 9.4^a^ |
| HS | 49.4 | 58.4 | 52.2 | 63.7 | 52.2 | 67.8 |
| >HS | 33.0 | 35.5 | 38.5 | 31.4 | 43.1 | 23.0 |
|  |  |  |  |  |  |  |
| Literacy, WRAT-3 score (X ± SE) | 41.0±0.4 | 41.7±0.3 | 44.1±0.3 | 39.5±0.3^a^ | 43.1±0.3 | 39.1±0.3^a^ |
| <36, % | 27.12 | 21.0^a^ | 15.8 | 29.5^a^ | 17.3 | 32.1^a^ |
| 37-40, % | 37.0 | 63.0 | 10.4 | 18.8 | 13.3 | 17.9 |
| 41-46,% | 23.4 | 28.4 | 21.3 | 29.9 | 26.3 | 26.2 |
| ≥47,% | 36.5 | 33.5 | 52.5 | 21.8 | 43.1 | 23.8 |
|  |  |  |  |  |  |  |
| % Unemployed in last month, yes | 31.7 | 40.7^a^ | 29.8 | 42.0^a^ | 22.2 | 56.3^a^ |
| % Unemployment in last month, missing | 17.7 | 17.1 | 24.8 | 11.9 | 25.2 | 7.0 |
|  |  |  |  |  |  |  |
| **Drug and tobacco use** |  |  |  |  |  |  |
| Any drug, current user, % | 21.5 | 11.7^a^ | 11.3 | 19.4^a^ | 12.8 | 20.2^a^ |
| Any drug, missing, % | 5.7 | 6.4 | 6.4 | 5.8 | 5.3 | 7.1 |
|  |  |  |  |  |  |  |
| Tobacco, current user, % | 50.3 | 40.2^a^ | 42.2 | 46.4 | 37.8 | 53.6^a^ |
| Tobacco, missing, % | 5.7 | 6.4 | 6.4 | 5.8 | 5.3 | 7.1 |
|  |  |  |  |  |  |  |
|  |  |  |  |  |  |  |
| **Self-rated health** |  |  |  |  |  |  |
| Poor, % | 4.4 | 4.5 | 5.5 | 3.7^a^ | 2.8 | 6.6^a^ |
| Average, % | 19.8 | 21.6 | 20.2 | 21.2 | 16.5 | 26.5 |
| Good, % | 40.6 | 40.3 | 36.8 | 43.1 | 41.6 | 38.9 |
| Very good, % | 26.4 | 26.0 | 28.8 | 24.2 | 28.8 | 22.6 |
| Excellent % | 8.8 | 7.6 | 8.7 | 7.7 | 10.2 | 5.4 |
|  |  |  |  |  |  |  |
| **% energy from grocery stores** |  |  |  |  |  |  |
| 100% | 27.6 | 30.0 | 24.9 | 32.0 ^a^ | 25.6 | 33.5^a^ |
| 50-99.9% | 58.4 | 57.3 | 60.6 | 55.7 | 59.2 | 55.9 |
| ≤50% | 14.1 | 12.7 | 14.6 | 12.3 | 15.3 | 10.6 |
|  |  |  |  |  |  |  |
|  |  |  |  |  |  |  |

^a^ P-value<0.05 based on *t*-test (continuous variables) or P-value<0.05 from χ2 test (categorical variables) for null hypothesis of no difference in means and proportions across sex, race or Poverty status. PIR=Poverty Income Ratio; SE=Standard Error; WRAT-3=Wide Range Achievement Test, version 3.
