# Supplementary material for: Monetary Value of Diet Is Associated with Dietary Quality and Nutrient Adequacy among Urban Adults, Differentially by Sex, Race and Poverty Status
Source: PLoS One. 2015 Nov 4;10(11):e0140905. doi: 10.1371/journal.pone.0140905 (PMC4633204; doi:10.1371/journal.pone.0140905)
Supplement: S2 Table — (DOCX) [file pone.0140905.s004.docx]

**S2 Table**. 2010-Healthy Eating Index and MAR/NAR scores by race and poverty status groups; HANDLS 2003-2009

|  | **2010-Healthy Eating Index (HEI)** | | |  |
| --- | --- | --- | --- | --- |
|  |  | | |  |
|  | **Whites** | **African-Americans** | **Above poverty** | **Below poverty** |
|  |  |  |  |  |
|  | X±SEM | X±SEM | X±SEM | X±SEM |
| HEI-2010 total score | 42.41±0.42 | 42.71±0.30 | 43.82±0.35^b^ | 40.93±0.34 |
| Total vegetables | 2.79±0.45 ^a^ | 2.58±0.04 | 2.73±0.04^b^ | 2.59±0.04 |
| Greens and beans | 0.89±0.05 ^a^ | 1.01±0.04 | 0.98±0.04 | 0.93±0.05 |
| Total fruit | 1.56±0.06 | 1.57±0.05 | 1.77±0.05^b^ | 1.30±0.05 |
| Whole fruit | 1.44±0.06 ^a^ | 1.02±0.04 | 1.42±0.05^b^ | 0.91±0.05 |
| Whole grains | 1.97±0.09 ^a^ | 1.67±0.07 | 1.96±0.07^b^ | 1.57±0.08 |
| Dairy | 4.46±0.09 ^a^ | 2.97±0.07 | 3.73±0.08^b^ | 3.42±0.09 |
| Total protein foods | 4.04±0.04 ^a^ | 4.42±0.02 | 4.25±0.03 | 4.27±0.03 |
| Seafood and plant proteins | 1.67±0.06 | 1.62±0.05 | 1.77±0.05^b^ | 1.47±0.05 |
| Fatty acids | 4.29±0.09 ^a^ | 5.57±0.08 | 5.06±0.08 | 4.99±0.09 |
| Sodium | 4.84±0.10 | 5.05±0.08 | 4.96±0.08 | 4.96±0.10 |
| Refined grains | 5.83±0.10 ^a^ | 6.52±0.08 | 6.18±0.08 | 6.29±0.09 |
| Empty calories | 8.64±0.19 | 8.71±0.15 | 9.01±0.16 ^b^ | 8.24±0.17 |
|  |  |  |  |  |
|  | **Mean adequacy ratio (MAR)** | | |  |
|  |  | | |  |
|  |  |  |  |  |
|  | **Whites** | **African-Americans** | **Above poverty** | **Below poverty** |
|  | X±SEM | X±SEM | X±SEM | X±SEM |
|  |  |  |  |  |
| MAR | 74.7±0.5 ^a^ | 71.6±0.4 | 73.4±0.5 | 72.2±0.5 |
| Vitamin A, NAR | 56.6±1.0 ^a^ | 50.0±0.8 | 53.4±0.9 | 51.9±1.0 |
| Vitamin C, NAR | 50.0±1.2 ^a^ | 54.3±1.0 | 54.8±1.0 ^b^ | 49.4±1.2 |
| Vitamin D, NAR | 25.4±0.8 ^a^ | 23.0±0.6 | 23.7±0.6 | 24.0±0.5 |
| Vitamin E, NAR | 43.4±0.8 ^a^ | 40.2±0.7 | 42.5±0.7 ^b^ | 40.2±0.8 |
| Vitamin B-6, NAR | 85.6±0.7 | 85.4±0.6 | 85.7±0.6 | 85.2±0.7 |
| Vitamin B-12, NAR | 92.4±0.6 ^a^ | 91.2±0.4 | 90.8±0.6 | 91.7±0.6 |
| Thiamin, NAR | 90.4±0.6 ^a^ | 85.2±0.6 | 87.9±0.6 | 86.8±0.7 |
| Riboflavin, NAR | 95.7±0.4 ^a^ | 92.3±0.4 | 93.9±0.4 | 93.5±0.5 |
| Niacin, NAR | 91.7±0.6 | 91.5±0.5 | 91.9±0.5 | 91.2±0.6 |
| Folate, NAR | 76.2±0.8 ^a^ | 69.8±0.7 | 73.2±0.7 | 71.6±0.8 |
| Iron, NAR | 80.1±0.8 | 78.1±0.7 | 79.5±0.7 | 78.2±0.8 |
| Copper, NAR | 90.6±0.6 | 89.6±0.5 | 90.5±0.5 | 89.4±0.6 |
| Zinc, NAR | 87.0±0.7 ^a^ | 83.7±0.6 | 84.7±0.6 | 85.6±0.7 |
| Calcium, NAR | 67.9±0.9 ^a^ | 58.0±0.7 | 63.7±0.8 ^b^ | 60.3±0.9 |
| Magnesium, NAR | 66.8±0.8 ^a^ | 59.3±0.6 | 63.6±0.7 ^b^ | 61.0±0.8 |
| Phosphorus, NAR | 95.9±0.4 ^a^ | 94.3±0.4 | 95.1±0.4 | 95.0±0.4 |
|  |  |  |  |  |

^a^ P<0.05 for null hypothesis of no difference by race in means of HEI-2010 (and components) and MAR/NAR scores, 2-sided independent samples t-test; ^b^ P<0.05 for null hypothesis of no difference by poverty status in means of HEI-2010 (and components) and MAR/NAR scores, 2-sided independent samples t-test.
